# Changing Clothing

**Published:** 1869-05

**Authors:** 


### CHANGING CLOTHING.

Many persons lose life every year by an injudicious change of clothing, and the principles involved need repetition almost every year.

If clothing is to be diminished, it should be done in the morning, when first dressing.

Additional clothing may be safely put on at any time.

In Northern States the under garments should not be changed for those less heavy sooner than the middle of May; for even in June a fire is very comfortable sometimes in a New York parlor.

Woolen flannel ought to be worn next the person, by all, during the whole year, but a thinner material may be worn after the first of June.

A blazing fire should be kept in every family room until ten in the morning, and rekindled again an hour before sundown, up to the first week in June, and from the first day of October.

Particular and tidy housekeepers by arranging their fireplaces for the summer too early, oftentimes put the whole family to a serious discomfort, and endanger health, by exposing them to sit in chilliness for several hours every morning, waiting for the weather to moderate, rather than have the fire-place or grate all blackened up; that is, rather than be put to the trouble of another fixing up for the summer, they expose the children to croup, and the old folks to inflammation of the lungs. The old and the young delight in warmth; it is to them the greatest luxury. Half the diseases of humanity would be swept from existence if the human body were kept comfortably warm all the time. The discomfort of cold feet, or of a chilly room, many have experienced to their sorrow; they make the mind peevish and fretful while they expose the body to colds and inflammations which often destroy it in less than a week.

